# Spatial and Temporal Biodiversity Patterns of Frugivorous Tephritid Communities Across Argentinean Drylands

**DOI:** 10.3390/insects17090895

**Published:** 2026-08-26

**Authors:** Segundo Ricardo Núñez-Campero, Flávio Roberto Mello Garcia, Lorena del Carmen Suárez, María Josefina Buonocore-Biancheri, Sergio Marcelo Ovruski

**Affiliations:** 1 Centro Regional de Investigaciones Científicas y Transferencia Tecnológica de La Rioja (CRILAR-CONICET), Entre Ríos y Mendoza s/n, Anillaco, La Rioja 5301, Argentina; segundo.nc@conicet.gov.ar; 2 Departamento de Ciencias Exactas, Físicas y Naturales, Instituto de Biología de la Conservación y Paleobiología, Universidad Nacional de La Rioja (UNLaR), Avenida Luis de la Fuente s/n, Ciudad de La Rioja, La Rioja 5300, Argentina; 3 Departamento de Ecologia, Zoologia e Genética, Instituto de Biologia, Universidade Federal de Pelotas, Pelotas 96000, Rio Grande do Sul, Brazil; 4 Dirección de Sanidad Vegetal, Animal y Alimentos de San Juan (DSVAA)-Gobierno de la Provincia de San Juan, Nazario Benavides 8000 Oeste, Rivadavia 5413, Argentina; lorenasuarez@conicet.gov.ar; 5 CCT CONICET San Juan, Av. Libertador Gral. San Martín 1109, San Juan J5400AR, Argentina; 6 Instituto y Museo de Ciencias Naturales, Facultad de Ciencias Exactas, Físicas y Naturales, Universidad Nacional de San Juan, Avenida España 406 Norte, San Juan J5400 DNQ, Argentina; sovruski@conicet.gov.ar; 7 Planta Piloto de Procesos Industriales Microbiológicos y Biotecnología (PROIMI-CONICET), Departamento de Control Biológico, Avda. Belgrano y Pje. Caseros, San Miguel de Tucumán 4000, Argentina; mjbuonocore@conicet.gov.ar

**Keywords:** tephritid fruit fly diversity, species assemblages, seasonal population dynamics, habitat segregation, *Anastrepha fraterculus*, *Ceratitis capitata*, *Rhagoletotrypeta*

## Abstract

Frugivorous fruit flies include several major agricultural pests, and understanding their species composition and seasonal abundance in natural environments is essential for improving management strategies. In Argentina, research has focused mainly on two economically important species, *Ceratitis capitata* and *Anastrepha fraterculus*, while native fruit flies in dryland ecosystems remain poorly studied. To address this gap, we conducted a year-long trapping survey in three dryland environments of northwestern Argentina: the dry Chaco, Chaco Montane Forest, and Monte, in order to characterize and compare community composition, diversity, species abundance, and temporal dynamics. We collected 1006 individuals representing seven native species and one exotic species. Community composition differed significantly among environments, showing strong spatial segregation. The native genus *Rhagoletotrypeta* dominated the overall catch and exhibited clear habitat preferences, with *R. xanthogastra* prevailing in the Dry Chaco and *R. pastranai* and *R. parallela* more abundant in the Monte and Chaco Montane Forest, respectively. The invasive *A. fraterculus* was the second most abundant species and the dominant species in Monte and Chaco Montane Forest, whereas *C. capitata* represented only a small fraction of the total catch. Fruit fly abundance peaked in late summer and autumn. These findings reveal strong habitat-driven structuring of fruit fly communities in drylands and provide ecological information that improves our understanding of tephritid assemblages in the region.

## Simple Summary

Fruit flies are important insects because some species damage fruit crops, while others play ecological roles that are still poorly understood. In Argentina, most studies have focused on two well-known pest species, but very little is known about the many native fruit flies that live in natural dryland environments. In this study, we monitored fruit flies for one full year in three dry ecosystems of northwestern Argentina. Our goal was to learn which species are present, how many individuals occur in each environment, and how their numbers change through the seasons. We collected 1006 fruit flies, most of them belonging to native species of a monophagous genus, *Rhagoletotrypeta*. Only a very small number belonged to the invasive Mediterranean fruit fly, a major global pest. We found that each environment had a different combination of species and that some native species were strongly associated with particular habitats. Another pest species, the South American fruit fly, showed a remarkable ability to thrive in all three environments. Fruit fly numbers increased toward the end of summer and during autumn. These findings improve our understanding of native fruit fly communities and provide information that can support better strategies for managing harmful species in the region.

## 1. Introduction

The “true” fruit flies (Diptera: Tephritidae) include 500 genera and more than 5000 named species, mostly distributed throughout the temperate, tropical, and subtropical regions of the world [1,2,3]. Ninety-five percent of tephritid species are phytophagous, as their immature stages require feeding on various plant tissues, such as fruits and vegetables, bamboo stems, flowers, and seeds [4]. Among all phytophagous tephritids, 40% are frugivorous and feed on healthy fruit still on the tree [5,6]. The females of these tephritid species lay their eggs deep inside the fruit, and these eggs develop into larvae, which feed on plant tissue and complete their life cycle in the soil as pupae enclosed in puparia [7,8,9]. There are approximately 250 known frugivorous fruit flies that cause serious damage to the agricultural economy, as their larvae attack the flesh and/or seeds of commercially important fruit crops [10,11,12]. The most damaging species belong to *Anastrepha* Schiner, *Bactrocera* Macquart, *Ceratitis* Macleay, *Rhagoletis* Loew, and *Zeugodacus* Hendel [13,14].

Analyzing the species composition and seasonal abundance of frugivorous tephritid fruit flies across diverse natural environments is essential for understanding community structures and temporal fluctuations [15,16,17,18]. Despite the ecological importance of these insects, the structure and seasonal behavior of tephritid communities in Argentine drylands remain largely undocumented, particularly in critical native xeric ecosystems such as the Chaco Árido and Monte. This study contributes to filling that gap by providing the first detailed description of tephritid fruit fly assemblages and their temporal patterns in these environments, helping distinguish between polyphagous species of economic relevance and native monophagous/stenophagous non-pest species within local communities. Additionally, this work offers a comparative framework for subsequent investigations into the underlying drivers of these dynamics.

Two approaches have conventionally been used to provide ecological information on fruit flies: (1) traps using food baits to monitor and estimate the abundance of adult fruit flies, and (2) systematic fruit sampling to determine the host range and quantify fruit infestation levels [16,19].

Within ecosystems, some frugivorous tephritid species are multivoltine and polyphagous—having a wide range of host plants from different families and not undergoing an apparent diapause—while others are univoltine and mono- or stenophagous, being limited to one or several host plant species within a particular genus and often undergoing diapause [20]. This latter group of species is highly sensitive to habitat loss due to their restricted diet, making them early warning indicators of environmental degradation or climate impacts [21]. Conversely, climate change, as well as the global trade in fresh fruits and vegetables, encourages invasions by multivoltine polyphagous species [22]. In this regard, studies on frugivorous tephritid ecology in Argentina have focused mainly on subtropical areas in which the African-native *Ceratitis capitata* (Wiedemann, 1824) and the Neotropical-native *Anastrepha fraterculus* (Wiedemann), two multivoltine polyphagous pest species, coexist [21]. These two species have major economic and quarantine significance worldwide [13]. Both pest species have been the subject of numerous ecological studies in Argentina on account of the damage and economic losses to fruit production [21,23,24,25]. Although 166 tephritid species have been reported in Argentina mainly through trapping between the 1950s and 1960s [26], studies on ecological interactions among frugivorous species of no economic importance are scarce [27]. However, research on this subject has intensified over the past 10 years. Ecological studies have been conducted on some mono- and stenophagous species of the genera *Rhagoletis*, *Haywardina*, and *Rhagoletotrypeta* (Trypetinae, Carpomyini: Carpomyina) [27,28,29,30,31,32] and the genus *Anastrepha* (Trypetinae, Toxotrypanini) [21,33].

Based on the facts outlined above and given the lack of research on tephritid diversity in arid and semiarid environments, a fruit fly survey was conducted using a sampling network over the course of a season in wild vegetation areas of three distinct dryland ecosystems of northwestern Argentina: the dry Chaco, Chaco Montane Forest, and the Monte Desert. Due to the dry conditions in such areas, fruit crops and domestic orchards rely on artificial irrigation, providing oases in rural and urban areas within a desert landscape where tephritid polyphagous pests, such as *C. capitata* and *A. fraterculus*, are highly able to thrive [24]. Given this background, it was hypothesized that: (1) the composition of the fruit fly tephritid community differs among the aforementioned dryland ecosystems; and (2) the structure of each tephritid community in such ecosystems is dominated by a few highly polyphagous invasive species, which relegate native species to lower abundance levels. Therefore, this study aimed to determine and compare the community structure of tephritid fruit flies in the three dryland ecosystems. In particular, species diversity and abundance, and the temporal dynamics of abundance by environment were examined. Achieving these aims will provide information not only on tephritid species assemblages, distributions, and temporal dynamics in wilderness areas, but also on habitat preferences. Furthermore, understanding the diversity of the local fruit fly community, including the population ecology of both exotic and native species, is a fundamental requirement for studying tephritid assemblages in natural and agricultural landscapes [34,35].

## 2. Materials and Methods

### 2.1. Study Area

**Figure 1:**
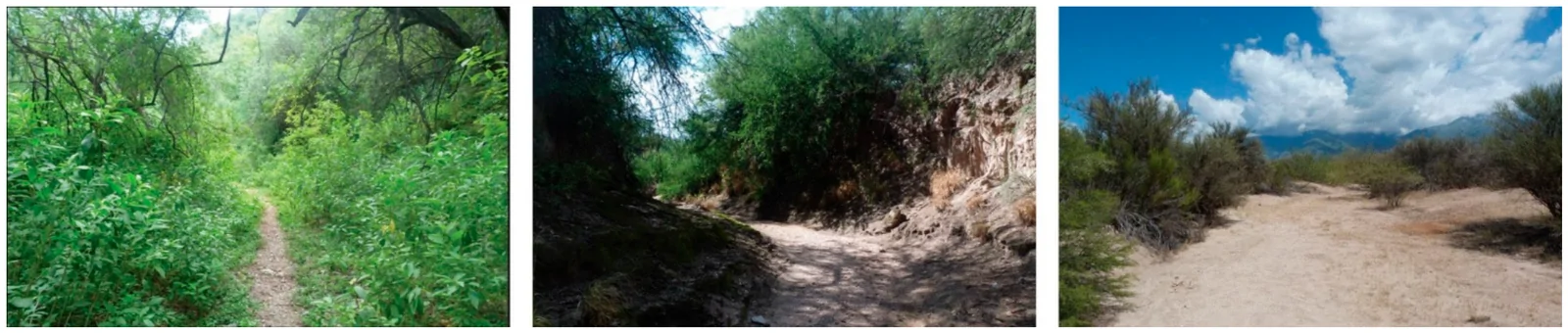
Landscapes of the three dryland environments sampled for tephritid fruit fly surveys in northwestern Argentina. From left to right: Chaco Montane Forest (CHF), Monte de Sierras y Bolsones (MONT), and Dry Chaco (DCH).

All collections were carried out in the province of La Rioja, northwestern Argentina. The study encompassed three well-differentiated environments: the Dry Chaco (DCH) and Chaco Montane Forest (CHF) districts, both belonging to the Chaco ecoregion, and the Monte de Sierras y Bolsones (MONT) ecoregion [36]. In this paper, the term “environment” is used generically to refer to these two districts of the Chaco and Monte ecoregions. Refer to Figure 1 for a visual overview of the environmental features of each site.

The Dry Chaco is a vast alluvial plain with a gentle eastward slope. Its climate is warm, subtropical and continental, with the highest absolute temperatures recorded in South America. Mean annual temperature decreases from north to south, ranging from 23 °C to 18 °C; mean summer temperatures often exceed 30 °C, while winter minima can drop to −16 °C [37]. Rainfall is markedly seasonal, concentrated from October to March, and varies from 500 to 700 mm per year, decreasing sharply towards the contact with the Monte [37] (Morello et al., 2012). The characteristic vegetation is a xerophytic forest dominated by *Schinopsis lorentzii* (Griseb.) Engl. (Anacardaceae), *Aspidosperma* Mart. and Zucc. (Apocynaceae), *Neltuma* spp. (ex-*Prosopis* spp.) (Fabaceae), *Bulnesia sarmientoi* Lorentz ex Griseb. (Zygophyllaceae), and numerous Cactaceae [37]. This forest becomes lower and more open in the southwestern, drier portion of the ecoregion, where a shrubby stratum becomes increasingly important [37].

The Chaco Montane Forest is found on the low foothills (900–1300 m) of the Sierra de Velasco, and its best representation can be observed in the ravines with eastern exposure (e.g., the ‘El Cantadero’ ravine). In low-slope areas, this district presents jungle-like aspects, with a tree stratum represented by *Parasenegalia visco* (Lorentz ex Griseb.) Seigler and Ebinger (Fabaceae) and *Lithraea molleoides* (Vell.) Engl. (Anacardaceae), which in some cases exceed 10–20 m in height. Both species are also present in the Dry Chaco and Monte, but here they undoubtedly form an exclusive community [36].

Some authors have suggested a possible mixed origin for this district, sharing fauna (avifauna) and vegetation [*Pisoniella arborescens* (Lag. and Rodr.) Standl. (Nyctaginaceae), *Cestrum lorentzianum* Griseb., *Vassobia breviflora* (Sendtn.) Hunz. (Solanaceae), *Kaunia lasiophthalma* (Griseb.) R.M.King and H.Rob. (Asteraceae), among others] with the Yungas, or subtropical forests of northwestern Argentina [38]. The unique characteristics of the Chaco Montane Forest and its location almost on the boundary between the Chaco and the Monte de Sierras y Bolsones ecoregions are the main reasons this district was selected for tephritid collection. The Monte de Sierras y Bolsones is a mountainous and basin-and-range region extending parallel to the Andes [37]. Its climate is arid to semi-arid, with a marked dry season and large daily thermal amplitude. Mean annual temperatures range from 12 °C to 18 °C, with summer maxima exceeding 35 °C and winter minima often below 0 °C. Precipitation is scarce, generally between 80 and 200 mm per year, and occurs mostly as summer thunderstorms; rainfall is slightly higher in the northern sector (up to 250 mm) and decreases southwards [37]. The landscape is dominated by extensive shrubby steppes, where the most widespread and characteristic species are *Larrea cuneifolia* Cav. and *L. divaricata* Cav. (Zygophyllaceae). Other common plants include *Bulnesia retama* (Gillies ex Hook. and Arn.) Griseb. (Zygophyllaceae), *Trichocereus terscheckii* (J.Parm. ex Pfeiff.) Britton and Rose (Cactaceae), and *Neltuma* spp., mainly in areas with access to groundwater or along watercourses. This vegetation is typically low, open, and highly adapted to water stress.

### 2.2. Insect Identification

The identification of the specimens was carried out by S.R.N.C. and S.M.O. using taxonomic keys [33,39,40,41,42]. The examined specimens were deposited in the entomological collection of CRILAR.

### 2.3. Experimental Design

Within each of the three environments described above, six sampling points separated by at least 95 m were established. One trap was placed by each point resulting in six permanent trapping sites per environment and a total of 18 sampling locations (see Table 1 for exact geographic coordinates and minimum intra- and inter-environment distances). At each site, a McPhail-type trap baited with hydrolyzed protein plus borax was suspended from the canopy of non-host natural vegetation (*Neltuma* spp., *Bulnesia retama*, *Schinopsis lorentzii*) at approximately 1.7–1.8 m above ground, thereby avoiding any bias that could arise from host plant preference. All traps remained continuously active throughout the entire sampling year, and they were serviced every two to four weeks (depending on climatic conditions) from early February 2023 to late January 2024, yielding 17 sampling periods (numbered 1 to 17). Although collection intervals differed, the sampling effort was constant. In each period, all captured tephritid flies were collected, preserved in 70% ethanol, and transported to the laboratory for identification as described above. The abundance of each species was recorded per trap and period. The study thus followed a repeated-measures design with two crossed factors: environment (three levels) and sampling period (17 levels), with trap sites as independent replicates nested within environments. The full dataset comprises 306 trap-period combinations (18 traps × 17 periods); periods with zero captures were retained for descriptive analyses but excluded from multivariate procedures where no community data existed.

### 2.4. Statistical Analyses

All statistical analyses were conducted using R version 4.3.2 [43]. Data manipulation and visualization were carried out with the packages *dplyr* v1.2.1 [44], *tidyr* v1.3.2 [45], *ggplot2* v4.0.3 [46], and *ggrepel* v0.9.6 [47]. The diversity and multivariate analyses were performed using the *vegan* v2.7-2 package [48]. Mixed-effects models were fitted with *lme4* v1.1-37 [49] and *glmmTMB* v1.1.13 [50]; overdispersion was checked using *performance* v0.15.1 [51]. Indicator species analysis was performed for all species with *indicspecies* v1.8.0 [52] but only statistically significant associations were reported in the results.

The required level of replication varied depending on the specific analysis. For multivariate analyses (PERMANOVA, NMDS), each trap–period combination was treated as an independent sample. For temporal abundance modeling (GLMM), each trap was considered an independent replicate and included as a random intercept. For alpha diversity analyses, data were aggregated at the environment–period level (see justification below).

Alpha diversity indices were computed at the environment–period level because diversity is an attribute of the community as a whole. Traps within an environment sample the same community and serve to increase spatial coverage rather than represent independent communities. Aggregating species counts by environment and sampling period avoids pseudo-replication and allows meaningful ecological comparisons among environments. Species abundances were first aggregated by environment and sampling period (hereafter “period”, a unique combination of date and trap collection). From these aggregates, four alpha diversity indices, species richness, Shannon diversity, Simpson diversity, and Pielou’s evenness were calculated using *vegan::specnumber* and *vegan::diversity*. To test for differences among environments, Kruskal–Wallis tests were applied, and the resulting *p*-values were adjusted for multiple comparisons (four indices) using the Bonferroni method. Effect sizes for alpha diversity indices were calculated as differences in mean values between environments, using summary statistics.

Beta diversity was explored by quantifying Bray–Curtis dissimilarities (*vegan::vegdist*). The differences in community composition among environments were assessed with PERMANOVA (*vegan::adonis2*) using two complementary models: one including both environment and period to account for temporal effects, and another using only environment to isolate the main spatial effect. Period was treated as a numeric covariate (Period_num) to increase parsimony. Each model was run with 9999 permutations. Pairwise comparisons among environments were conducted using the pairwise.adonis2 function from *pairwiseAdonis* v0.4.1 [53] with 9999 permutations. For each comparison, both unadjusted *p*-values and *p*-values corrected for multiple testing using the Benjamini–Hochberg FDR method were reported. The homogeneity of multivariate dispersion was tested using *betadisper* followed by permutation tests, which helped validate the PERMANOVA assumptions. Ordination was carried out using non-metric multidimensional scaling (NMDS) with two dimensions (*vegan::metaMDS*). To visualize the contribution of each taxon, species scores were plotted as arrows originating from the origin of the ordination space. Temporal changes in total abundance were analyzed with generalized linear mixed models (GLMMs), and because count data contained many zeros, three error structures were compared: Poisson, negative binomial, and zero-inflated Poisson. Overdispersion and zero inflation were assessed through this model comparison, which allowed selecting the distribution that best accommodated the data. All models included environment and period (as a numeric covariate for parsimony) as fixed effects and trap site as a random intercept, reflecting the independence of traps within each environment (minimum separation ≥ 95 m) and accounting for repeated measures over time. The best-fitting model was selected based on the lowest Akaike Information Criterion (AIC). Once the optimal model was chosen, its coefficients were extracted and reported.

To evaluate whether the temporal parametrization influenced model outcomes, we additionally fitted GLMMs treating sampling period as a categorical factor (17 levels), while retaining trap identity as a random intercept. Likewise, PERMANOVA models were re-run with period entered as a categorical factor. These alternative models were used to assess the robustness of the temporal effects and to determine whether the main conclusions depended on the representation of time. Because the categorical-period models produced results consistent with those obtained using the numeric covariate, and the numeric formulation provided greater parsimony and model stability, the numeric covariate was retained in the main analyses. Full outputs of the categorical-period models are provided in Supplementary File S1.

Sampling completeness was evaluated by generating species accumulation curves using the specaccum function in the *vegan* package, with 100 random permutations. Curves were constructed for the entire dataset and separately for each ecoregion, including all sampling periods (i.e., all combinations of ecoregion and collection period, regardless of whether captures were zero). This approach accounts for the full sampling effort (17 periods × 3 ecoregions = 51 samples) and allows the assessment of inventory completeness even in the presence of frequent zero-capture periods.

To complement the accumulation curves with quantitative completeness metrics, we calculated Chao1 richness and Good’s sample coverage for each sampling unit. Chao1 was computed from the frequency of rare species (singletons and doubletons), and sample coverage was calculated as Good’s coverage (1 − F1/N), where F1 is the number of singletons and N the total abundance.

To provide measures of uncertainty for the mean within- and between-environment Bray–Curtis dissimilarities, bootstrap resampling (999 iterations) was used to estimate standard errors.

## 3. Results

### 3.1. Species Abundance Patterns

**Figure 2:**
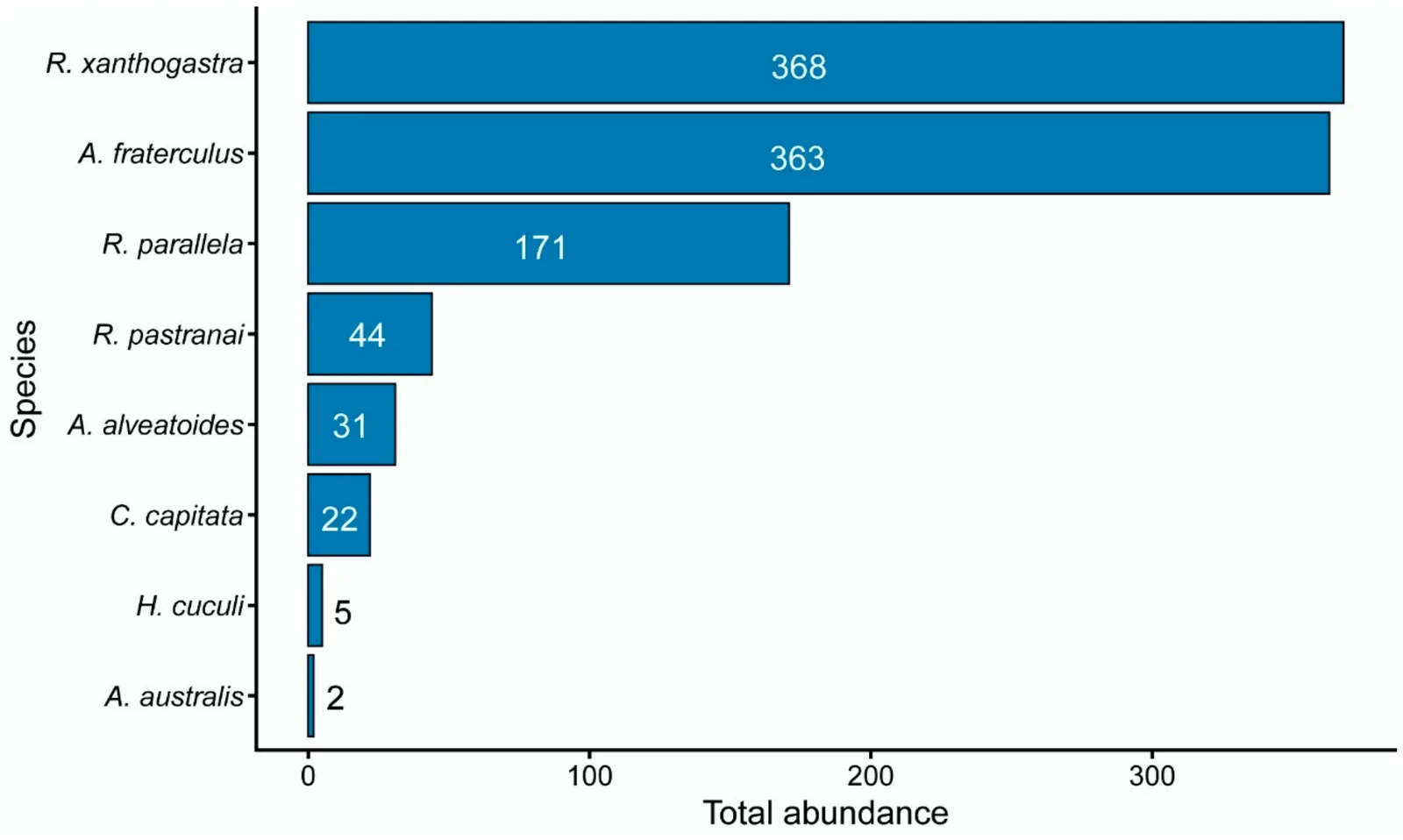
Total abundance (number of individuals) of each tephritid species collected across all sampling periods and environments. Species are ordered from highest to lowest total abundance.

**Figure 3:**
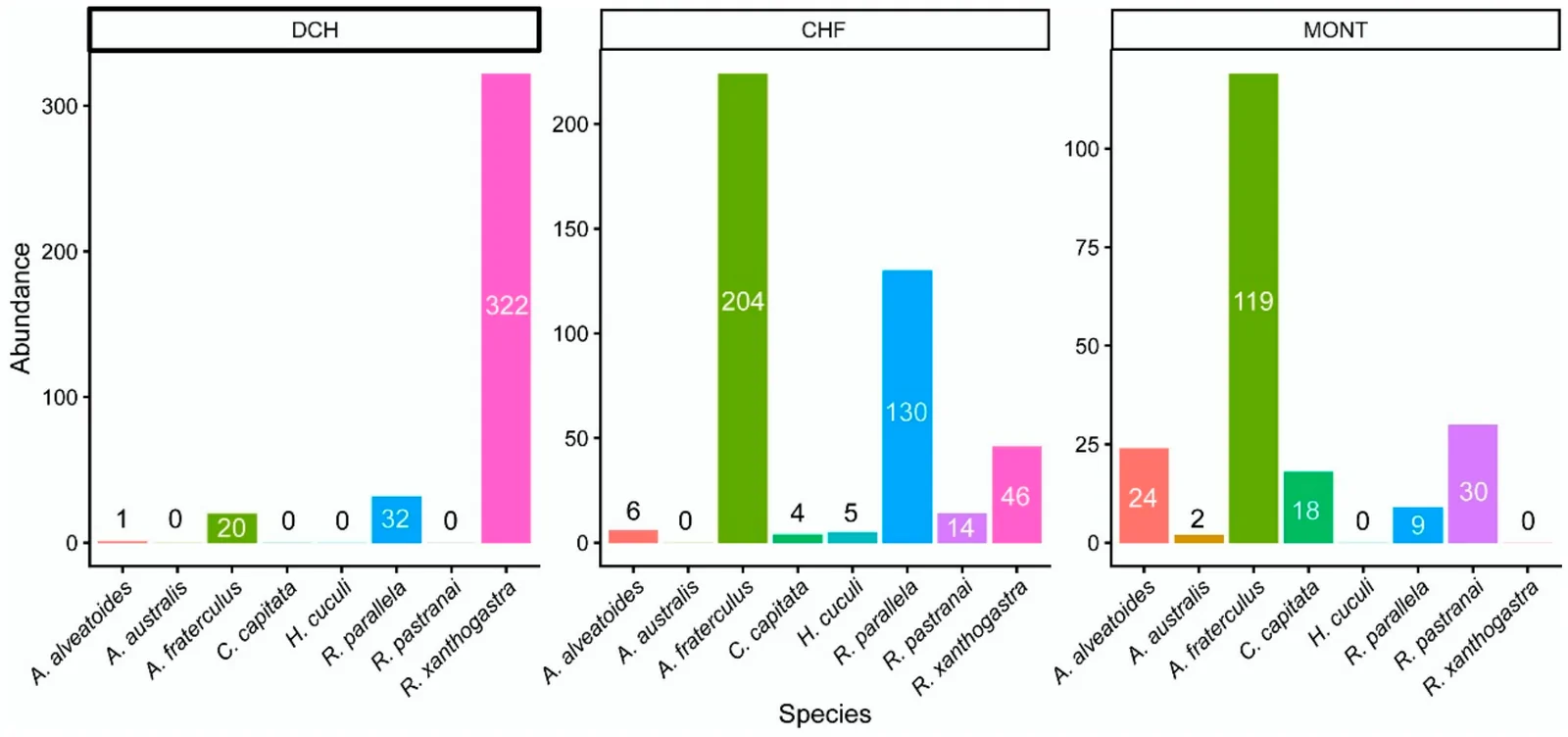
Species abundance by environment. Each panel (facet) represents one environment (DCH = Dry Chaco, CMF = Chaco Montane Forest, MONT = Monte). Bars show the total number of individuals of each species collected in each environment. The x-axis scales vary among panels to optimize visualization of small counts.

Seven native and one exotic tephritid species were caught: *Rhagoletotrypeta xanthogastra* Aczél, 1951, *R. pastranai* Aczél, 1954, *R. parallela* Norrbom, 1994, *Haywardina cuculi* (Hendel, 1914), *Anastrepha alveatoides* (Blanchard, 1961), *A. australis* (Blanchard, 1960), *A. fraterculus* (natives for the neotropics), and *Ceratitis capitata* (exotic). Both *R. xanthogastra* and *A. fraterculus* were the most abundant species, which together accounted for more than 70% of all tephritids collected (Figure 2). *Rhagoletotrypeta xanthogastra* was the most abundant tephritid, but it was mainly found in the dry Chaco, accounting for nearly 90% of the total catch (Figure 3). *Anastrepha fraterculus* occurred in all three environments tested but was most prevalent in both the Chaco Montane Forest and Monte (Figure 3).

The Chaco Montane Forest exhibited the highest overall abundance, with *A. fraterculus* and *R. parallela* as co-dominants. The Monte, in turn, showed intermediate abundance and was characterized by the prominence of *A. fraterculus* and *R. pastranai* (Figure 3).

### 3.2. Temporal Dynamics of Abundance

**Figure 4:**
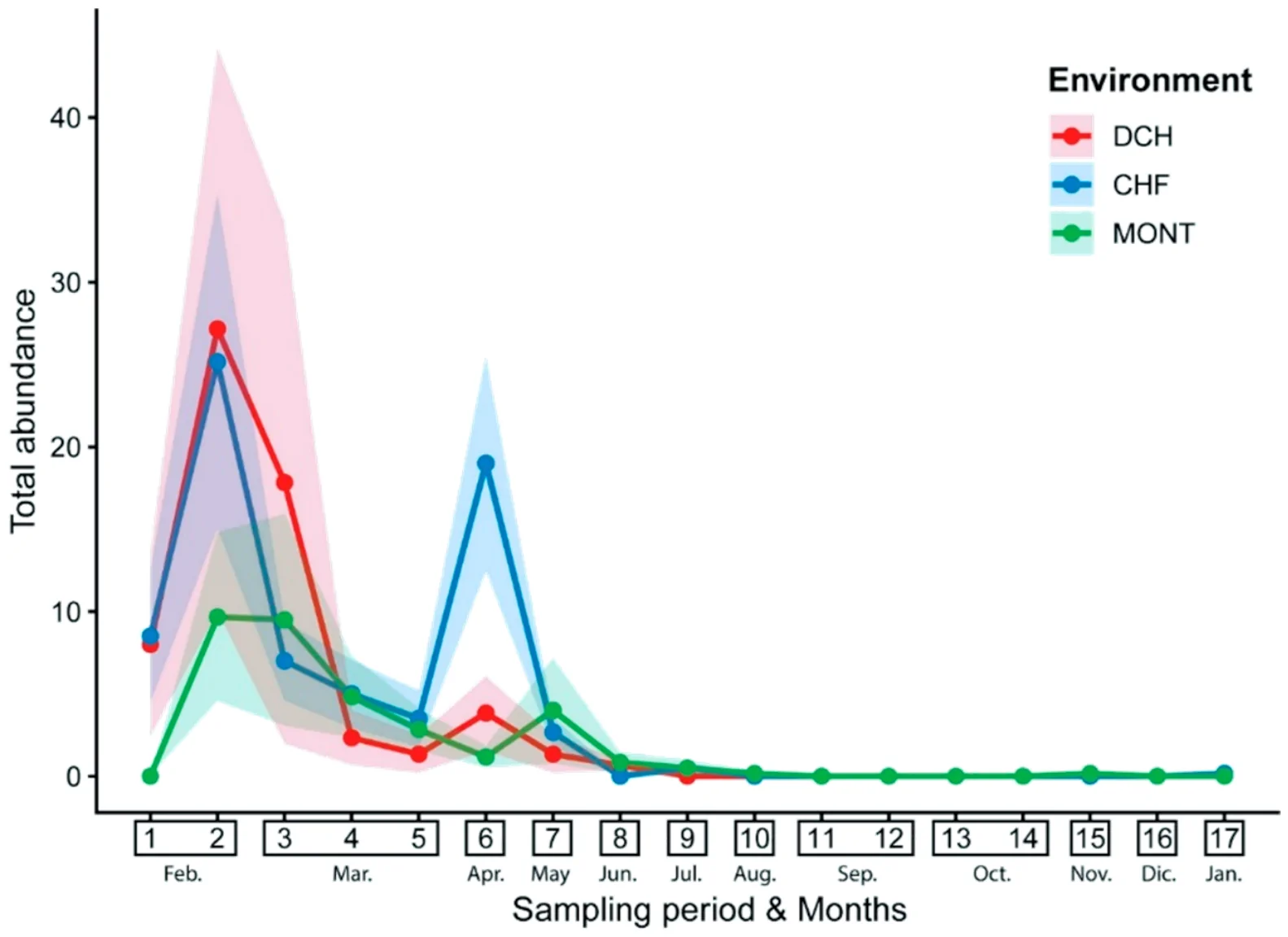
Temporal dynamics of total abundance across the 17 sampling periods (February 2023 to January 2024). The solid line represents the mean, and the shaded ribbons indicate ±1 *SE* (standard error). Means and standard errors were calculated for each trapping site, sampling period, and environment.

Temporal patterns revealed a pronounced and synchronous seasonal pulse across all environments. Abundance peaked sharply during February and March (summer), followed by a rapid decline toward July (winter), when catch levels dropped to zero or close to zero (Figure 4). The Chaco Montane Forest exhibited the most complex temporal abundance pattern, with a strong secondary increase in April (autumn) (Figure 4). The dry Chaco showed a similar abundance pattern, but there was a sharper decline in catches after the first population peak (Figure 4). The Monte showed a more attenuated seasonal variation, with a second population peak in autumn, though not at the same time as that found in the other two environments (Figure 4).

The GLMM with the negative binomial distribution model provided the best fit, with an AIC of 794.0, compared to 2471.7 for the Poisson and 1667.1 for the zero-inflated Poisson. This indicates that the negative binomial model adequately handled overdispersion and that no residual zero inflation remained after model fitting. This model confirmed a significant decline in total abundance over the sampling period (*β* = −0.544, *SE* = 0.051, *z* = −10.72, *p* < 0.001; 95% *CI* = −0.644 to −0.444), reflecting a consistent seasonal decrease. Compared to the dry Chaco, the Chaco Montane Forest had significantly higher abundance (*β* = 0.861, *SE* = 0.403, *z* = 2.14, *p* = 0.033; 95% *CI* = 0.071 to 1.651), whereas the Monte did not differ significantly (*β* = 0.232, *SE* = 0.428, *z* = 0.54, *p* = 0.588; 95% *CI* = −0.607 to 1.071). The random intercept variance among trap sites was 0.302 (*SD* = 0.55).

Because the temporal dataset is extensive, detailed numerical values for each sampling period are provided in Supplementary File S2. The main text focuses on the seasonal trends, which are more clearly visualized in Figure 4.

### 3.3. Alpha Diversity

Alpha diversity varied across environments and sampling periods, but the within-environment temporal fluctuations (as reflected by the standard deviations in Table 2) were sufficiently large to mask any statistically significant differences among environments (Bonferroni-adjusted *p* > 0.05 for all indices). The Chaco Montane Forest consistently exhibited the highest richness and diversity values, followed by the Monte, whereas the dry Chaco showed the lowest diversity. Although these trends were not statistically significant, the magnitude of the differences suggests ecologically meaningful variation. Mean richness in the Chaco Montane Forest exceeded that of the dry Chaco by 1.56 species, and the Monte showed an intermediate increase of 0.62 species relative to the dry Chaco. Similar patterns were observed for Shannon diversity, with the Chaco Montane Forest and Monte exceeding the dry Chaco by 0.28 and 0.20 units, respectively. Simpson diversity also showed modest positive differences (0.09) for both CHF and MONT relative to DCH.

**Figure 5:**
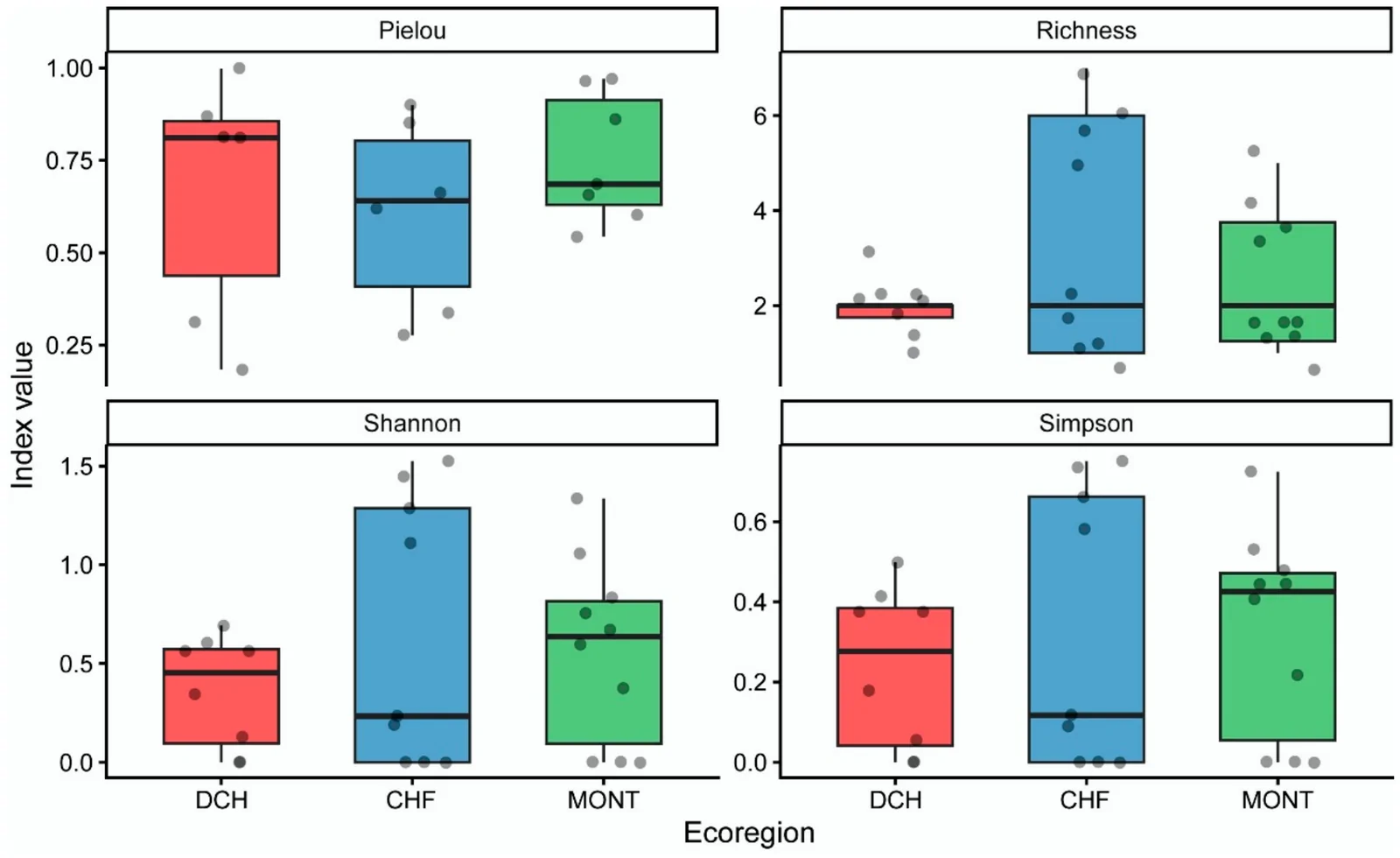
Alpha diversity indices (Richness, Shannon, Simpson, Pielou) by environment. Box plots display the median, interquartile range (box), and range (whiskers) of index values across sampling periods with non-zero total abundance. Points (jittered) show individual period values. No significant differences were detected among environments after Bonferroni correction (Kruskal–Wallis tests, adjusted *p* > 0.05 for all indices).

Despite these ecological trends, Kruskal–Wallis tests followed by Bonferroni correction (for four indices) detected no significant differences among environments for richness (*χ*^2^ = 1.04, *p*_adj_ = 1.000), Shannon diversity (*χ*^2^ = 0.84, *p*_adj_ = 1.000), Simpson diversity (*χ*^2^ = 0.66, *p*_adj_ = 1.000), or Pielou’s evenness (*χ*^2^ = 0.72, *p*_adj_ = 1.000). To complement these analyses, sampling completeness was quantified using Chao1 and sample coverage estimates. Chao1 values were nearly identical to observed richness, and sample coverage was consistently high (≥0.93 in most sites), indicating that the majority of species present were detected (complete results in Supplementary File S3). The complete alpha diversity dataset is available in Supplementary File S4, while summary statistics are shown in Table 2 and the distribution of indices is visualized in Figure 5.

**Figure 6:**
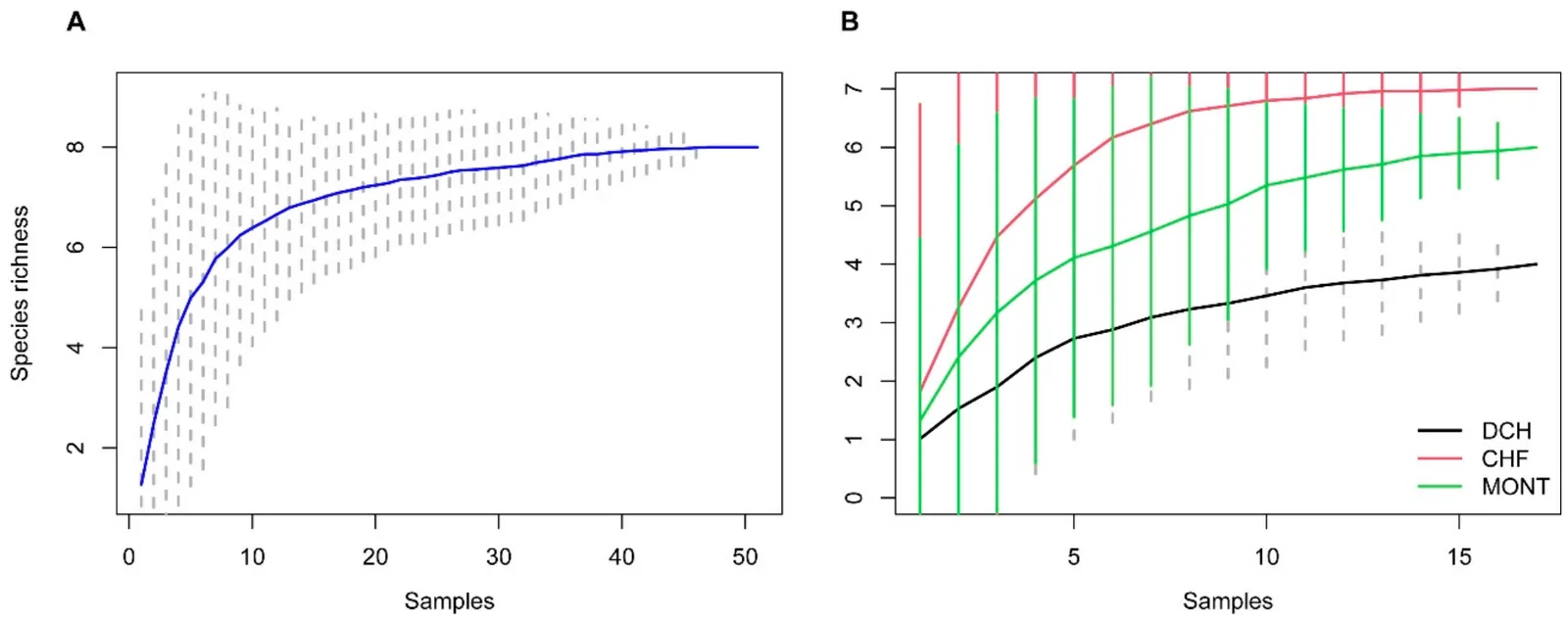
Species accumulation curves based on 100 random permutations. (**A**) Overall cumulative curve for all samples combined. (**B**) Cumulative curves for each environment separately. Vertical lines represent the 95% confidence intervals derived from the standard deviation among permutations.

The species accumulation curves approached an asymptote at approximately eight species for the overall dataset (Figure 6A). The curves for individual environments showed a similar pattern, albeit with different saturation rates: the dry Chaco curve saturated rapidly, whereas the Monte and Chaco Montane Forest curves saturated more slowly (Figure 6B).

### 3.4. Beta Diversity and Community Structure

**Figure 7:**
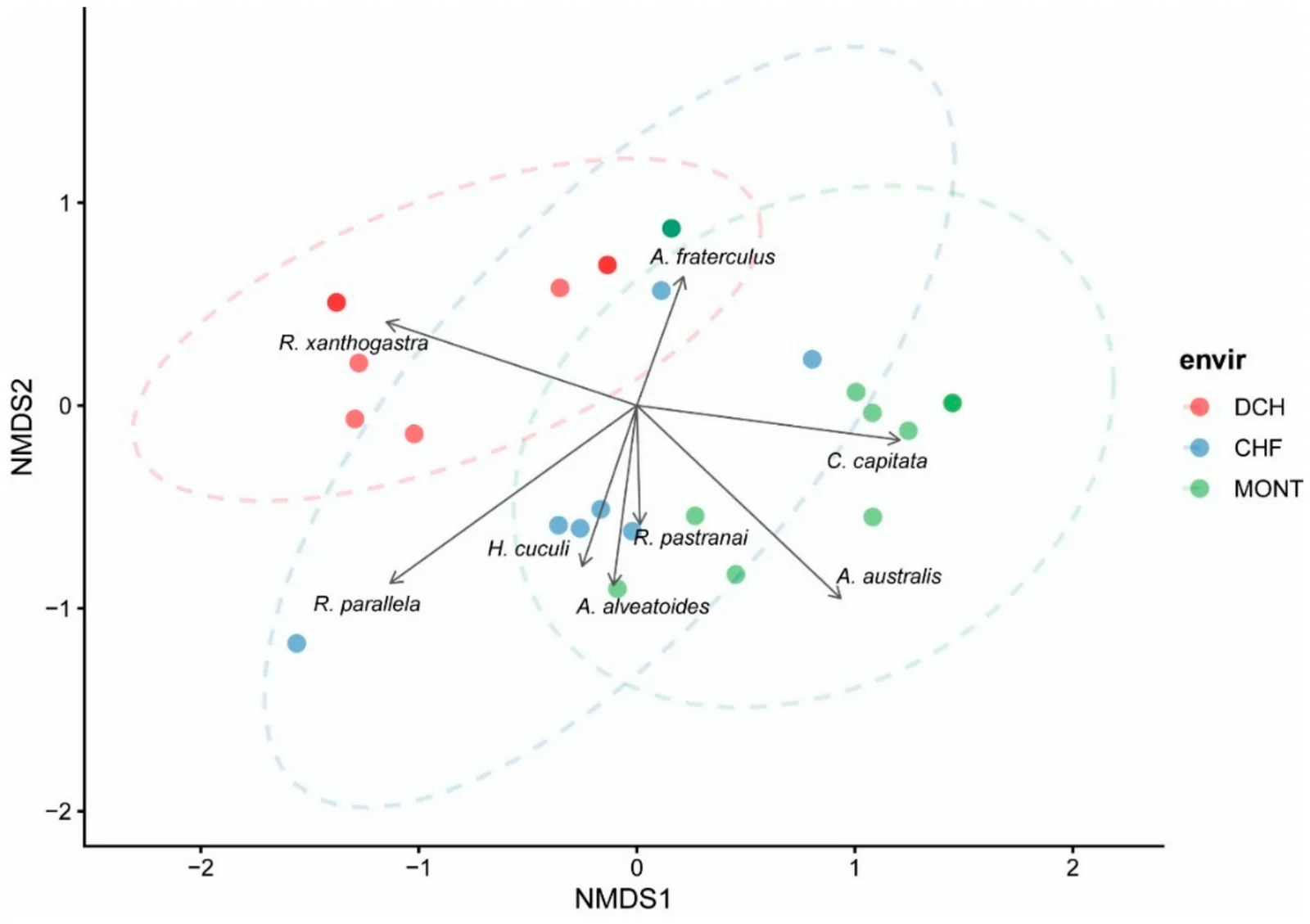
Non-metric multidimensional scaling (NMDS) ordination of tephritid communities based on Bray–Curtis dissimilarities (stress = 0.08, indicating excellent representation). Points represent individual sampling periods, colored by environment (red = dry Chaco, blue = Chaco Montane Forest, green = Monte). Dashed ellipses indicate the multivariate dispersion of sampling periods within each environment, illustrating the degree of within-group variability. Species scores are plotted as arrows from the origin; the direction and length of each arrow indicate the contribution of that species to the ordination.

The NMDS ordination (stress = 0.08) showed distinct clustering of tephritid species by environment (Figure 7). Species found in the dry Chaco formed a tight cluster driven by the dominance of *R. xanthogastra* (see arrow pointing to that group in Figure 7). Species occurring in the Chaco Montane Forest were more dispersed and occupied an intermediate position in the ordination space, with substantial overlaps with both the dry Chaco and the Monte. In contrast, species recorded from the Monte formed a more compact and clearly separated cluster toward the lower right. The arrows pointing to the tephritid species showed the contribution of each taxon: *R. xanthogastra* pointed toward the dry Chaco cluster, *A. fraterculus* toward the Chaco Montane Forest, while *C. capitata* and *A. australis* pointed toward Monte.

**Figure 8:**
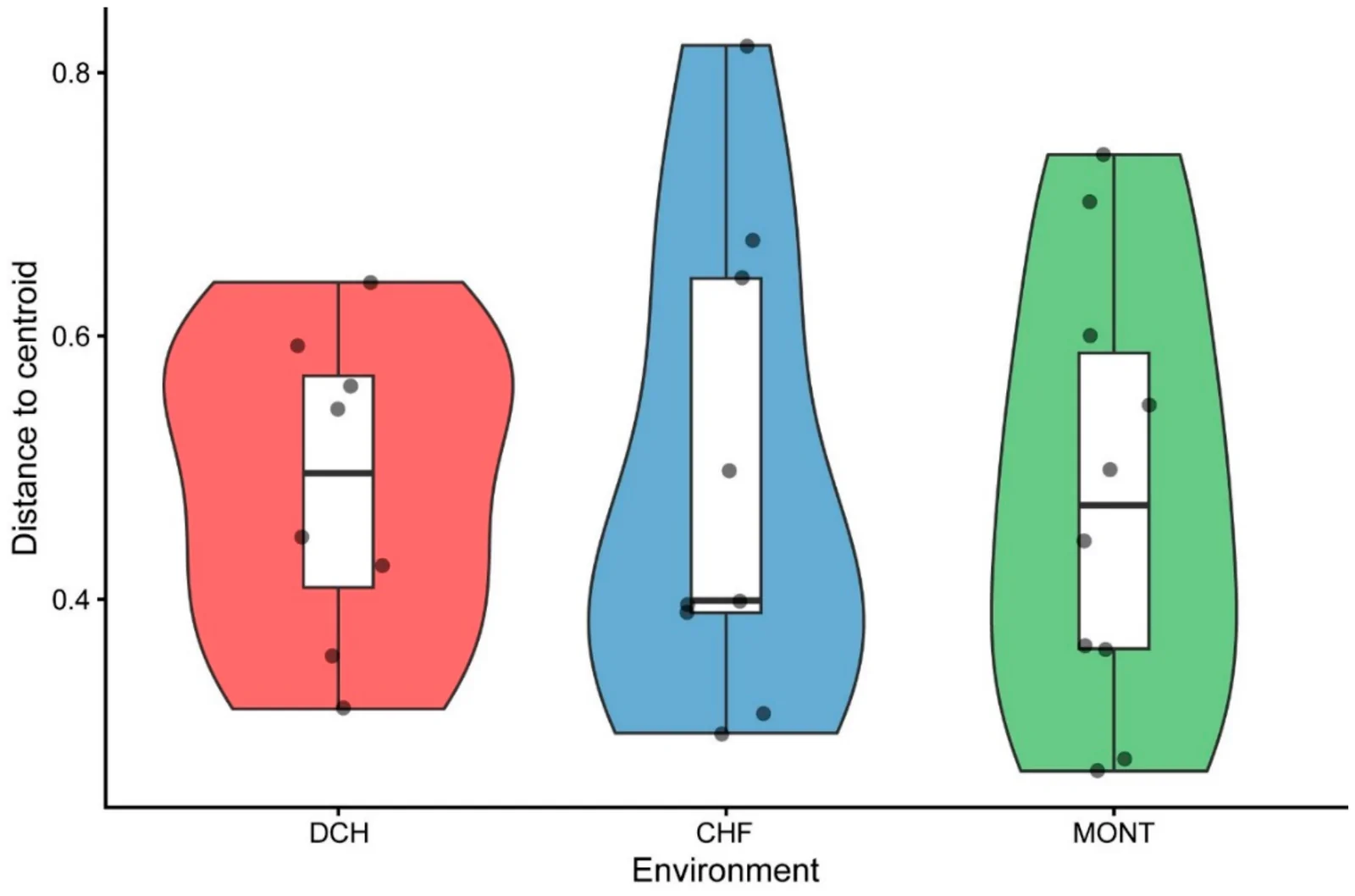
Multivariate dispersion (distance to centroid) for each environment based on Bray–Curtis distances. Violin plots show the distribution of distances, with box plots (median and quartiles) overlaid. The permutation test for homogeneity of dispersions was not significant (*F* = 0.013, *p* = 0.986), confirming that the PERMANOVA results reflect true compositional differences rather than differences in within-group variability.

Multivariate analyses revealed clear and statistically significant differences in the species composition of the tephritid community among environments. PERMANOVA with environment as the sole predictor (Bray–Curtis distance, 9999 permutations) explained 22.0% of the variation in species composition (*F* = 3.39, *p* = 0.0003). When the sampling period was included as a covariate, the combined model explained 70.3% of the variance (*F* = 2.37, *p* = 0.0001). Pairwise comparisons indicated that community composition differed significantly between dry Chaco and Chaco Montane Forest (*F* = 2.85, *R*^2^ = 0.160, *p* = 0.012, *p_adj_* = 0.017) and between dry Chaco and Monte (*F* = 5.46, *R*^2^ = 0.255, *p* < 0.001, *p_adj_* = 0.001), whereas the difference between Chaco Montane Forest and Monte was not significant (*F* = 1.97, *R*^2^ = 0.104, *p* = 0.058, *p_adj_* = 0.060). Multivariate dispersion did not differ among environments (*F* = 0.013, *p* = 0.986), indicating that group dispersions were statistically homogeneous according to PERMDISP (Figure 8).

To summarize spatial turnover, Table 3 presents within- and between-environment Bray–Curtis dissimilarities. Within-environment dissimilarity was high in all cases (0.708–0.742), indicating substantial internal heterogeneity. Between-environment dissimilarity was even higher, particularly between dry Chaco and Monte (0.902), highlighting strong species turnover across the landscape.

Indicator species analysis identified three significant associations: *R. xanthogastra* was strongly associated with the dry Chaco (IndVal = 0.942, *p* = 0.0001), *A. fraterculus* with the Chaco Montane Forest and Monte (IndVal = 0.888, *p* = 0.0125), and *C. capitata* with the Monte (IndVal = 0.801, *p* = 0.0029). No other species showed a significant indicator value.

## 4. Discussion

The species composition and community structure of tephritid fruit flies were recorded for the first time in three different dryland environments of northwestern Argentina, namely the dry Chaco, Chaco Montane Forest, and the Monte. In this regard, the results of the current study provide valuable ecological insights, as follows: (1) tephritid communities differed consistently among the ecosystems studied, with each environment hosting a distinct fruit fly assemblage characterized by its particular diversity and seasonal abundance patterns; (2) a relatively similar pattern in the seasonal abundance of tephritid species across different environments, with population peaks in summer and autumn, although with a particular trait of the Chaco Montane Forest; (3) the high occurrence in all three environments of a highly polyphagous, multivoltine, invasive species native to the South American Neotropical wetlands, such as *A. fraterculus*; (4) the predominance of *A. fraterculus* over other species within the tephritid community in both Monte and Chaco Montane Forest; (5) the occurrence of another highly polyphagous, multivoltine invasive species, though at low abundance, such as the exotic *C. capitata* in both Monte and Chaco Montane Forest; and 6) the predominance of the non-economically important native genus *Rhagoletotrypeta* among the overall number of trapped tephritids and the apparent environment associations among the identified species.

Although differences in species occurrence among ecoregions may suggest non-random patterns, our sampling design, based exclusively on adult captures in baited traps, does not allow formal inference of habitat preference or specialization. Consequently, these patterns indicate environmental associations or differential occurrence rather than definitive habitat preferences.

The first finding revealed that differences in fruit fly community composition among environments were statistically significant, as demonstrated by the significant PERMANOVA results and the clear spatial segregation revealed by the NMDS clustering. Because multivariate dispersion among environments was homogeneous, these patterns reflect genuine differences in community structure rather than differences in variability among sampling units. These findings strongly support the hypothesis that landscape features play a key role in shaping tephritid community structure. The marked differences among the environments studied, particularly between the dry Chaco and Monte ecoregions, indicated substantial species turnover across the landscape. In this regard, the results showed the native *R. xanthogastra* as a highly dominant tephritid species only in the dry Chaco, a finding confirmed by the analysis of indicator species. This pattern suggests that local environmental conditions and specific host plants in the dry Chaco may influence this tephritid species’ occurrence, a hypothesis warranting future study. Global studies on tephritid communities propose that strong habitat-specific constraints—such as microclimate, vegetation structure, and host-plant phenology—often shape fruit fly assemblages, and our results provide a baseline for evaluating whether similar mechanisms operate in these native dryland ecosystems [13,16,54,55]. Such a pattern is consistent with the concept of beta diversity driven by environmental gradients, where changes in vegetation structure, host plant availability, and microclimate lead to distinct faunal assemblages [56,57,58]. The Chaco Montane Forest’s intermediate position in the clustering space clearly reflects its status as a transition zone. This environment primarily harbored a mix of species identified in the adjacent environments, dry Chaco and Monte, as well as particular species from the Chaco Montane Forest, such as *H. cuculi*. In turn, the highest overall tephritid abundance in the Chaco Montane Forest is congruent with the concept that transition habitats typically display high resource heterogeneity, which supports mixed faunas [59,60,61,62,63]. The Chaco Montane Forest consistently showed higher diversity and abundance than the dry Chaco and the Monte, but without a significant difference from the latter. This lack of significance has ecological relevance, as insect communities in arid and semi-arid systems typically exhibit strong fluctuations driven by constant changes in resource availability, extreme temperatures, and high rainfall variability [64,65]. In this regard, it is important to note that multivariate dispersion did not differ between environments, meaning that the PERMANOVA results reflected a true ecological divergence between environments rather than differences in variability within the tephritid communities.

Concerning the second finding, the GLMM confirmed a significant negative effect of sampling period on total tephritid abundance, reflecting a consistent seasonal decrease after a sharp population peak in late summer in all tested environments. Although a second population peak was recorded in the fall in all three habitats, this peak was particularly pronounced in the dry Chaco Montane Forest. This may suggest higher resource availability or broader ecological niches compared to the other habitats, as the Chaco Montane Forest is characterized by high biodiversity [60]. Such prolonged periods of activity have been documented in transitional habitats where phenological overlap among plant species extends resource periods [66]. The sharp decline in the dry Chaco following its first peak in summer could reflect more restrictive climatic conditions or a more constrained phenology of host plants. These temporal patterns validate the hypothesis that tephritid communities exhibit strong seasonal dynamics, and this could be driven by climatic and phenological cycles characteristic of arid and semi-arid ecosystems [67]. This temporal beta diversity is characteristic of insect communities in seasonal environments and reflects the interaction between climatic constraints and the phenology of resources [68,69]. In addition to climatic and phenological drivers, other ecological and methodological factors may also contribute to the observed seasonal patterns. For example, variation in host fruit availability and differences in fruiting phenology among dominant plant species could influence the magnitude and timing of abundance peaks. Likewise, interspecific interactions, such as competition for oviposition sites or resource partitioning, may modulate the temporal dynamics of particular species. Methodological aspects, including potential trap attractiveness biases or seasonal changes in detectability, could also affect capture rates; however, because all traps used the same attractant and were deployed simultaneously across environments, any such bias would be consistent across sampling sites and unlikely to generate the observed ecological differences. These unexamined factors are plausible explanations but require further confirmation. Despite the spatial and temporal patterns documented in the present study, several limitations should be noted. First, the species accumulation curves approached an asymptote for both the overall dataset and each habitat individually when all sampling periods (including those with zero captures) were considered. Nevertheless, the high proportion of zero-capture periods likely influenced the shape of the curves, and the possibility of undersampling during periods of low insect activity cannot be ruled out. Therefore, while the asymptote suggests that the observed richness is representative, the true species richness may be underestimated due to the seasonal dormancy of the community. Second, the study was limited to a single year due to budgetary constraints on scientific research in Argentina; therefore, interannual variability could not be assessed. In arid and semi-arid ecosystems, consecutive years often exhibit marked differences in temperature and precipitation regimes, which can substantially affect insect phenology, host plant availability, and population dynamics [64]. Consequently, long-term monitoring would be necessary to determine whether the observed patterns are consistent over the years or whether they reflect the particular climatic conditions of the sampling year.

The third to fifth findings highlighted the high ability of both invasive polyphagous pest species, *A. fraterculus* and *C. capitata*, to spread throughout Argentina and adapt to wild areas with extreme environmental conditions, such as the drylands surveyed in this study. Interestingly, *A. fraterculus* was the second most abundant tephritid species, accounting for 36% of the total number of individuals trapped across all three environments. In addition, *A. fraterculus* was the dominant species in both the Monte and Chaco Montane Forest environments, accounting for 59% and 52% of the total trapped species, respectively. In turn, *A. fraterculus* clearly outnumbered the stenophagous *A. australis*, previously classified under *Toxotrypana* Gerstaecker (Norrbom et al. 2018), and associated with *Morrenia* L. (Apocynaceae) [33,70,71], *A. alveatoides* (first time recorded for La Rioja province), associated with *Ximenia americana* L. (Olacaceae) [62,72]. These data are particularly relevant for two main reasons: (1) *A. fraterculus*, one of the most economically significant fruit flies in Argentina [24], can thrive in environments outside its usual habitats, such as warm, humid subtropical areas of northwestern and northeastern Argentina, in which this tephritid species is mostly restricted [23]; and (2) well-established *A. fraterculus* populations in arid/semi-arid natural habitats underscore their high level of polyphagy in exploiting wild host plants, which may perform as major reservoirs for the infestation of fruit crops located in isolated, irrigated fruit-producing areas of northwestern and central-western Argentina. These two events may highlight the high degree of ecological plasticity of *A. fraterculus*, as evidenced by its broad host range, highly competitive ability, and environmental adaptability [73,74,75], traits that characterize invasive and dominant tephritids [12,76]. In this regard, even though *A. fraterculus* comprises a group of cryptic species distributed throughout the Neotropical region [77], in Argentina there is only one valid species with different populations suited to local conditions [78]. In contrast, the exotic *C. capitata*, another invasive pest species of quarantine significance [12], was considerably less abundant than *A. fraterculus*, accounting for only 2.2% of the total number of caught tephritid species. Interestingly, *C. capitata* was only the fourth most abundant tephritid species in Monte, but in the Chaco Montane Forest, it was the least frequently found species. This information may suggest a more restricted distribution and lower adaptability of *C. capitata* within environments with native wild vegetation, at least in dryland environments. This is consistent with distribution records suggesting that *C. capitata* populations mainly persisted on uncontested exotic host plants and thrived in landscapes highly degraded by human activity [79,80,81,82,83]. Current research suggests that native monophagous or stenophagous *Anastrepha* species, alongside native parasitoids, may actively exclude *C. capitata* from preserved patches of native vegetation [21,84]. Nevertheless, although *C. capitata* was found in native dryland areas, albeit at very low levels of abundance, this is a significant indicator that some native hosts may be serving as reservoirs for this dangerous pest. For example, the native *X. americana*, which is widely distributed in the dry Chaco and Monte ecoregions, has previously been recorded as a host of *C. capitata* in La Rioja [23]. The second hypothesis was partially supported by the results, considering the *A. fraterculus* dominance in two of the three environments studied; nevertheless this was not the case for the other invasive pest *C. capitata*.

The sixth finding disclosed a high incidence of the genus *Rhagoletotrypeta*, close to 60% of all caught tephritids. This native fruit fly genus, closely associated with hackberries (*Celtis* L., Cannabaceae) [27,41], was represented by three species belonging to the *xanthogastra* group. Interestingly, data on the abundance levels of the three *Rhagoletotrypeta* species relative to the tested environments suggest differential occurrence across environments for each species. In this regard, *R. xanthogastra* was not only the most frequently caught species of all tephritids, but it also had a strong prevalence in the dry Chaco, accounting for 91% of the trapped individuals of the genus *Rhagoletotrypeta*. In turn, *R. pastranai* and *R. parallela* predominantly occurred in the Monte and the Chaco Montane Forest, accounting for 77% and 69% of all individuals of the genus, respectively. Although *R. pastranai* is the only species for which there is extensive knowledge regarding its life-history strategies [32], only limited ecological information is available about the other two species, such as host plant–fly–parasitoid interactions and distribution ranges in Argentina [27,30]. Similar to this study, previous data also recorded *R. xanthogastra* in the dry Chaco ecoregion of northwestern Argentina, whereas *R. parallela* was also found in a transition environment between the dry Chaco and the southernmost sector of the subtropical Yungas forests of northwestern Argentina [27]. In contrast to findings of the current study, *R. pastranai* has previously been recorded in transition environments between the dry Chaco and the southernmost and northernmost subtropical Yungas of northwestern Argentina [32], and in xerophytic forests in northeastern Buenos Aires [30]. However, *R. pastranai* had not been previously recorded from both the Monte ecoregion, with its extreme xeric conditions, and the Chaco Montane Forest between the dry Chaco and Monte ecoregions. Based on earlier information and the current data, *R. pastranai* may have a broader range of environmental tolerance than *R. xanthogastra* and *R. parallela*. However, long-term ecological studies are needed to confirm this assertion and to evaluate the processes underlying these patterns. Even so, our results provide an important baseline for understanding the diversity and temporal fluctuations of tephritid communities in the Chaco and Monte environments of La Rioja, offering a foundation upon which future ecological and biogeographical research can build.

## 5. Conclusions

The findings of this study reveal distinct spatial assemblages and pronounced temporal dynamics in tephritid communities across the tested dryland ecosystems of northwestern Argentina. These patterns are consistent with hypotheses proposed in global studies, which suggest that habitat-specific factors, such as environmental features, resource phenology, and seasonal climatic variation, could plausibly influence fruit fly assemblages. Although our data do not allow evaluating these mechanisms directly, the patterns documented here provide a baseline for future studies aimed at testing these ecological processes. In this regard, the results support the first hypothesis that the composition of the tephritid community varies across different tested environments, and the seasonal abundance peaks may reflect associations with these particular fruit fly complexes. The second hypothesis was partially verified, as the tephritid community in two of the three dryland ecosystems, Monte and Chaco Montane Forest, was dominated by the multivoltine polyphagous *A. fraterculus*. This invasive pest species showed patterns consistent with environmental plasticity, enabling it to exploit and persist in these conserved natural habitats. However, *A. fraterculus* faced higher resistance to invasion in the dry Chaco ecosystem, which could be associated with increased competition with species of the native genus *Rhagoletotrypeta*, which inhabit drought-resistant xerophytic forests. Even though the invasive polyphagous species *C. capitata* was recorded with very low occurrence rates, its presence in the studied environments is indicative of its high environmental plasticity. This study provides a better understanding of the ecology of native tephritid flies in xeric habitats and offers baseline information on the diversity and temporal dynamics of fruit fly communities in the region.

## Figures and Tables

**Table 1:** Geographic coordinates and spatial arrangement of the 18 trapping locations across the three environmental units surveyed: Dry Chaco (DCH), Chaco Montane Forest (CHF; a systemic complex within the Dry Chaco region), and Monte de Sierras y Bolsones (MONT). Columns indicate: Environment, the environmental unit where each trap was placed; Trap (1–6) assigned to individual traps within each environment; lat and lon, geographic coordinates in decimal degrees; alt, elevation in meters above sea level; Min. Dist. Intra (km), the minimum distance from each trap to another trap within the same environment; and Min. Dist. Inter (km), the minimum distance from each trap to the nearest trap located in a different environment.

Environment	Trap	lat	lon	alt	Min. Dist. Intra (km)	Min. Dist. Inter (km)
DCH	1	−29.31	−66.76	422	0.095	14.063
DCH	2	−29.31	−66.76	427	0.095	14.137
DCH	3	−29.3	−66.75	426	0.163	12.842
DCH	4	−29.3	−66.75	426	0.163	12.991
DCH	5	−29.19	−66.67	431	0.106	12.825
DCH	6	−29.19	−66.67	430	0.106	12.781
CHF	1	−29.17	−66.81	855	0.147	12.928
CHF	2	−29.17	−66.8	872	0.147	12.781
CHF	3	−29.18	−66.82	875	0.157	13.842
CHF	4	−29.18	−66.82	885	0.157	13.976
CHF	5	−29.19	−66.81	858	0.057	12.862
CHF	6	−29.2	−66.81	847	0.057	12.842
MONT	1	−28.8	−66.9	1246	4.976	42.634
MONT	2	−28.8	−67	1702	4.465	45.586
MONT	3	−28.86	−66.92	1354	5.469	37.038
MONT	4	−28.83	−66.97	1568	4.465	41.152
MONT	5	−28.76	−66.93	1290	2.433	47.387
MONT	6	−28.75	−66.95	1373	2.433	49.298

**Table 2:** Summary statistics (mean ± SD) of alpha diversity indices by environment. Indices were calculated for each sampling period with a non-zero total abundance. Richness = number of species; Shannon = Shannon diversity index; Simpson = Simpson diversity index; Pielou = Pielou’s evenness (Shannon/ln(Richness)). No significant differences among environments were found after Bonferroni correction (Kruskal–Wallis tests, adjusted *p* > 0.05 for all indices).

Environment	Richness	Shannon	Simpson	Pielou
DCH	1.88 ± 0.64	0.36 ± 0.28	0.24 ± 0.20	0.66 ± 0.33
CHF	3.44 ± 2.51	0.64 ± 0.68	0.33 ± 0.34	0.61 ± 0.26
MONT	2.50 ± 1.43	0.56 ± 0.47	0.33 ± 0.26	0.76 ± 0.18

**Table 3:** Within- and between-environment Bray–Curtis dissimilarities. Within type values are the mean dissimilarities among sampling periods of the same environment; between type values are the mean dissimilarities between-environment periods of different environments. Higher dissimilarities between environments indicate strong species turnover across the landscape.

Comparison	Bray–Curtis	Type
Within-DCH	0.714	Within
Within-CHF	0.742	Within
Within-MONT	0.708	Within
DCH–CHF	0.819	Between
DCH–MONT	0.902	Between
CHF–MONT	0.765	Between

## Data Availability

The raw data presented in this study are available on the CONICET (Argentina) website link: http://hdl.handle.net/11336/286109 (accessed on 29 March 2026).

## Supplementary Materials

The following supporting information can be downloaded at: https://www.mdpi.com/article/10.3390/insects17090895/s1, File S1: Alternative temporal parametrizations used in GLMM and PERMANOVA. File S2: Total abundance (sum of all species) by ecoregion and sampling period. File S3: Richness estimators and sample coverage for each sampling unit. File S4: Alpha diversity indices (richness, Shannon, Simpson, Pielou) for each ecoregion and sampling period with non-zero total abundance.
