# Supplementary material for: Spatial and Temporal Biodiversity Patterns of Frugivorous Tephritid Communities Across Argentinean Drylands
Source: Insects. 2026 Aug 26;17(9):895. doi: 10.3390/insects17090895 (PMC13607389; doi:10.3390/insects17090895)
Supplement: Supplementary file 1 [file insects-17-00895-s001.zip › File S1.pdf]

Supplementary File S1

Alternative Temporal Parametrizations in GLMM and PERMANOVA

Overview

To assess the robustness of the temporal effects reported in the main manuscript, we fitted additional models treating the sampling period as a categorical factor rather than a numeric covariate. These analyses evaluate whether the conclusions depend on the parametrization of time and provide complementary evidence supporting the main results.

1. GLMM with Period as a Categorical Factor

Model specification

A negative binomial GLMM was fitted:

$$\text{Total abundance} \sim \text{Environment} + \text{Period (categorical)} + (1 \mid \text{Trap})$$

The model included 17 period levels, allowing non-linear seasonal patterns.

Model selection

Comparison of Poisson and negative binomial models indicated that the negative binomial GLMM remained the best-supported model:

- AIC (Poisson): 1970.66
- AIC (Negative Binomial): 784.98

This matches the distributional choice in the main manuscript.

Key results

Environment effects

- **CHF vs. DCH:** Estimate = 0.6387, $p = 0.0637$ (higher abundance, marginal significance)
- **MONT vs. DCH:** Estimate = 0.2565, $p = 0.4625$ (no significant difference)

These results are consistent with the GLMM using a numeric temporal covariate.

Temporal effects

The categorical period factor recovered the expected seasonal decline:

- Early periods showed higher abundance (e.g., Period 2: Estimate = 1.403, $p = 0.0132$).
- Mid- and late-season periods showed significantly lower abundance:
  - Period 8: Estimate = –2.334, $p = 0.0004$
  - Period 9: Estimate = –2.978, $p = 4.7\times10^{-5}$
  - Period 10: Estimate = –4.665, $p = 8.7\times10^{-5}$
  - Period 15: Estimate = –4.560, $p = 6.7\times10^{-5}$
  - Period 17: Estimate = –4.745, $p = 1.1\times10^{-4}$

Several late-season periods (11–14) produced unstable estimates with extremely large standard errors due to very low or zero counts across traps.

Interpretation

The categorical period GLMM confirms the main temporal pattern: a strong seasonal decline in abundance. The ecological interpretation of Environment effects remains unchanged. The numeric covariate used in the main manuscript provides a more parsimonious and stable representation of the temporal trend, avoiding collinearity and overparameterization.

2. PERMANOVA with Period as a Categorical Factor

Model specification

A PERMANOVA using Bray–Curtis dissimilarities was fitted:

$$\text{Community composition} \sim \text{Environment} + \text{Period (categorical)}$$

An interaction model was also attempted.

Key results

Additive model (Environment + Period)

Environment + Period: $Df = 13$, $R^2 = 0.703$, $F = 2.369$, $p = 0.0002$

Interpretation: Environment remains the dominant source of variation in community structure. Period explains additional temporal variation but does not alter spatial segregation.

Interaction model (Environment × Period)

The interaction model was saturated: $R^2 = 1$, No meaningful F or p values could be computed.

This is expected given the large number of period levels relative to sample size.

Interpretation

The PERMANOVA with categorical period corroborates the main findings: community composition differs significantly among Environments, and this spatial structure is robust to alternative temporal parametrizations. Temporal variation is detectable but does not modify the primary spatial patterns.

3. Summary

Both alternative analyses—GLMM and PERMANOVA with period as a categorical factor—support the conclusions presented in the main manuscript:

- The effect of Environment on abundance and community composition is robust.
- The seasonal decline in abundance is consistently recovered across models.
- The numeric temporal covariate used in the main text is justified on grounds of parsimony, model stability, and interpretability.
- The categorical-period models provide complementary evidence and are included here for transparency.
