# Supplementary material for: Spatial and Temporal Biodiversity Patterns of Frugivorous Tephritid Communities Across Argentinean Drylands
Source: Insects. 2026 Aug 26;17(9):895. doi: 10.3390/insects17090895 (PMC13607389; doi:10.3390/insects17090895)
Supplement: Supplementary file 1 [file insects-17-00895-s001.zip › File S2.pdf]

Supplementary File S2

Total abundance (sum of all species) by ecoregion and sampling period. Zero values indicate no captures during that period.

Ecoregion	Sampling period	Total abundance
DCH	1	48
**DCH**	2	163
DCH	3	107
**DCH**	4	14
DCH	5	8
**DCH**	6	23
**DCH**	7	8
**DCH**	8	4
**DCH**	9	0
**DCH**	10	0
**DCH**	11	0
**DCH**	12	0
**DCH**	13	0
**DCH**	14	0
**DCH**	15	0
**DCH**	16	0
**DCH**	17	0
**CHF**	1	51
**CHF**	2	151
**CHF**	3	42
**CHF**	4	30
**CHF**	5	21
**CHF**	6	114

**CHF**	7	16
**CHF**	8	0
**CHF**	9	3
**CHF**	10	0
**CHF**	11	0
**CHF**	12	0
**CHF**	13	0
**CHF**	14	0
**CHF**	15	0
**CHF**	16	0
**CHF**	17	1
**MONT**	1	0
**MONT**	2	58
**MONT**	3	57
**MONT**	4	29
**MONT**	5	17
**MONT**	6	7
**MONT**	7	24
**MONT**	8	5
**MONT**	9	3
**MONT**	10	1
**MONT**	11	0
**MONT**	12	0
**MONT**	13	0
**MONT**	14	0
**MONT**	15	1
**MONT**	16	0

**MONT**	17	0
