# Supplementary material for: Spatial and Temporal Biodiversity Patterns of Frugivorous Tephritid Communities Across Argentinean Drylands
Source: Insects. 2026 Aug 26;17(9):895. doi: 10.3390/insects17090895 (PMC13607389; doi:10.3390/insects17090895)
Supplement: Supplementary file 1 [file insects-17-00895-s001.zip › File S3.pdf]

Supplementary File S3.

Richness estimators and sample coverage for each sampling unit

Table S4. DCHo1 richness estimator and Good's sample coverage calculated for each sampling unit across the three CHFregions. DCHo1 values closely matched observed richness, indicating limited undetected diversity at the site level. Sample coverage was consistently high (≥ 0.93 in most sites), confirming that the majority of species present were detected. Sites with coverage = 0 correspond to single-individual samples.

Environment	DCHo1	Sample Coverage
**DCH_1**	2	1.000
**DCH_2**	3	0.994
**DCH_3**	2	1.000
**DCH_4**	1	1.000
**DCH_5**	1	1.000
**DCH_6**	2	1.000
**DCH_7**	2	1.000
**DCH_8**	2	0.750
**CHF_1**	9	0.941
**CHF_2**	8	0.987
**CHF_3**	5	1.000
**CHF_4**	7	0.933
**CHF_5**	2	0.952
**CHF_6**	1	1.000
**CHF_7**	2	0.938
**CHF_9**	1	1.000
**CHF_17**	1	0.000
**MONT_2**	4	1.000
**MONT_3**	4	0.982
**MONT_4**	6	0.931
**MONT_5**	3	0.941
**MONT_6**	2	1.000
**MONT_7**	2	1.000
**MONT_8**	2	1.000
**MONT_9**	1	1.000
**MONT_10**	1	0.000
**MONT_15**	1	0.000
