# Supplementary material for: Spatial and Temporal Biodiversity Patterns of Frugivorous Tephritid Communities Across Argentinean Drylands
Source: Insects. 2026 Aug 26;17(9):895. doi: 10.3390/insects17090895 (PMC13607389; doi:10.3390/insects17090895)
Supplement: Supplementary file 1 [file insects-17-00895-s001.zip › File S4.pdf]

Supplementary File S4

Alpha diversity indices (richness, Shannon, Simpson, Pielou) for each ecoregion and sampling period with non-zero total abundance. "—" indicates that Pielou's evenness is undefined for richness = 1.

Ecoregion	Sampling period	Richness	Shannon	Simpson	Pielou
**DCH**	1	2	0.604	0.413	0.871
**DCH**	2	3	0.344	0.178	0.313
**DCH**	3	2	0.128	0.055	0.184
**DCH**	4	1	0.000	0.000	—
**DCH**	5	1	0.000	0.000	—
**DCH**	6	2	0.692	0.499	0.999
**DCH**	7	2	0.562	0.375	0.811
**DCH**	8	2	0.562	0.375	0.811
**CHF**	1	6	1.112	0.583	0.621
**CHF**	2	7	1.287	0.662	0.661
**CHF**	3	5	1.447	0.736	0.899
**CHF**	4	6	1.525	0.751	0.851
**CHF**	5	2	0.191	0.091	0.276
**CHF**	6	1	0.000	0.000	—
**CHF**	7	2	0.234	0.117	0.337
**CHF**	9	1	0.000	0.000	—
**CHF**	17	1	0.000	0.000	—
**MONT**	2	4	1.335	0.725	0.963
**MONT**	3	4	0.836	0.446	0.603
**MONT**	4	5	1.056	0.530	0.656
**MONT**	5	3	0.753	0.443	0.685
**MONT**	6	2	0.598	0.408	0.863
**MONT**	7	2	0.377	0.219	0.544

**MONT**	8	2	0.673	0.480	0.971
**MONT**	9	1	0.000	0.000	—
**MONT**	10	1	0.000	0.000	—
**MONT**	15	1	0.000	0.000	—
